# Modeling the Local Dynamics of Cardiovascular Health: Risk Factors, Context, and Capacity

**Published:** 2008-03-15

**Authors:** Diane R Orenstein, Jack Homer, Bobby Milstein, Kristina Wile, Parakash Pratibhu, Rosanne Farris

**Affiliations:** Applied Research and Evaluation Branch, Division for Heart Disease and Stroke Prevention, National Center for Chronic Disease Prevention and Health Promotion, Centers for Disease Control and Prevention; Homer Consulting, Voorhees, New Jersey; Division of Adult and Community Health, National Center for Chronic Disease Prevention and Health Promotion, Centers for Disease Control and Prevention, Atlanta, Georgia; Sustainability Institute, Stow, Massachusetts; Association of Schools of Public Health, Atlanta, Georgia; Applied Research and Evaluation Branch, Division of Heart Disease and Stroke, National Center for Chronic Disease Prevention and Health Promotion, Centers for Disease Control and Prevention, Atlanta, Georgia

## Project Background and Rationale

Broad strategies for reducing the burden of heart disease and stroke in the United States are identified in *A Public Health Action Plan for Heart Disease and Stroke Prevention* (1), the American Heart Association's Community Guidance (2), and other policy documents (3). These guides call for a spectrum of interventions extending from primordial prevention to end-of-life care, but they do not specify how best to allocate limited resources. Not only are data on effect sizes lacking for certain intervention pathways, but the contributing risk factors and social determinants interact in dynamically complex ways that defy simple calculation.

System dynamics modeling is a methodology for better anticipating the likely effects of interventions in dynamically complex situations. It has been successfully applied since the 1970s to many areas of public health and social policy, including cardiovascular disease (4,5). The methodology is used to 1) map the most salient features that contribute to a persistent problem; 2) convert the map into a computer simulation model suitable for comparing alternative policy scenarios; 3) compare results from simulated experiments to identify intervention strategies that might plausibly alleviate the problem; and 4) conduct sensitivity analyses to assess areas of uncertainty in the model and guide future research.

The Centers for Disease Control and Prevention's (CDC's) Division for Heart Disease and Stroke Prevention, in collaboration with the Sustainability Institute and Research Triangle Institute, has embarked on a system dynamics modeling project to better understand trends in cardiovascular health at a local level and to evaluate potential intervention strategies. The project focuses on the prevention and management of risk factors among those who have never experienced a cardiovascular event rather than on the care of people afterward. That is, we concentrate on the "upstream" challenge of minimizing risk rather than on the better understood "downstream" task of postevent care.

Major risk factors for cardiovascular disease include hypertension, high cholesterol, diabetes, smoking, obesity, poor diet, inadequate physical activity, psychosocial stress, secondhand smoke, and air pollution (6). The combined effects of interventions addressing these risk factors are not clearly understood, in part because of complex causal relationships among the risk factors and in part because of differences from place to place in contextual factors that affect those relationships. These contextual factors include social determinants of health, such as neighborhood safety, as well as local policy, environmental, and institutional conditions, such as smoking regulations, transportation options, and community organizing. One step toward better anticipating the potential for place-based interventions is to create a dynamic model that can help us understand causal relationships diagrammatically and also assess their behavior over time. This essay discusses an emerging framework for studying the dynamics of cardiovascular risk in context, one that is now being translated into a quantified model that can be tested through simulation experiments.

## Addressing Variations in Local Context

Contemporary public health work increasingly considers the importance of place, and by extension, the local contextual factors that may affect people's health status or their responses to perceived problems (7-9). Understanding these place-based factors affecting health is necessary for crafting effective intervention strategies (10,11).

The notion of local context is complicated by its many dimensions, which can include factors as diverse as differential access to goods and services, established cultural norms, inequalities in socioeconomic position, racism, chronic stress, political power, and neighborhood infrastructure. It is difficult to define such conditions conceptually and virtually impossible to perform controlled real-world experiments to understand their individual and combined contributions to cardiovascular risk. As a consequence, local contextual factors are often excluded when planning and evaluating policies or programs.

## Collaborating With a Local Partner

An early decision in our modeling strategy was to partner with colleagues based in a small city who could share insights and data about their particular conditions, information that would help us to develop a model that indicates how local context affects cardiovascular risk. After a national search, we joined forces with a team from Austin/Travis County, Texas, on the basis of their rich understanding of the health, behavioral, socioeconomic, and political trends in the area. This team participated in the CDC's Steps to a HealthierUS (STEPS) program, which has allowed it to gather a wide spectrum of data focusing on the eastern part of the county, where incomes are lower and the burden of disease is higher than on the west side.

## An Emerging Framework

A conceptual scheme, shown in the Figure, forms the basis for our simulation modeling of cardiovascular health in context. Like other such schemes (12), it specifies pathways by which social and physical conditions may affect cardiovascular health. This framework will continue to evolve as we learn more from prior research and from our colleagues in Austin. Costs (top right) are an important endpoint for intervention analysis. These include the direct medical costs and the costs of lost productivity for first-time cardiovascular events and deaths as well as the costs for other medical complications attributable to the risk factors of hypertension, diabetes, smoking, and obesity.

**Figure 1.**
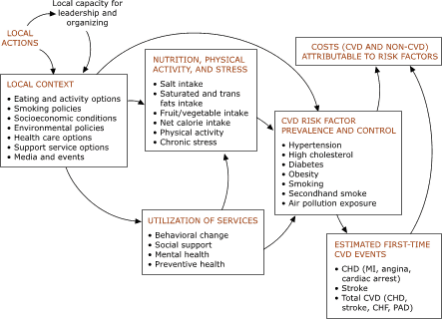
A policy framework for cardiovascular risk in context

**Table:** 

The figure is a flowchart that begins with local actions; this element includes eating and activity options, smoking policies, socioeconomic conditions, environmental policies, health care options, support service options, and media and events. These local actions lead to local capacity for leadership and organizing, which further reinforces the local actions. The local actions element also leads to cardiovascular disease (CVD) risk factor prevalence and control; nutrition, physical activity, and stress; and utilization of services.
CVD risk factors include hypertension, high cholesterol, diabetes, obesity, smoking, secondhand smoke exposure, and air pollution exposure. The element of CVD risk factor prevalence and control leads to costs (CVD and non-CVD) attributable to risk factors and estimated first-time events, such as coronary heart disease (myocardial infarction, angina, cardiac arrest), stroke, and total cardiovascular disease (coronary heart disease, stroke, congestive heart failure, peripheral arterial disease). These events also contribute to the costs attributable to risk factors.
The element of nutrition, physical activity, and stress takes into account such factors as salt intake, bad fats intake, fruit/vegetable intake, net calorie intake, physical activity, and chronic stress. This element also affects CVD risk factor prevention and control. The element of utilization of services — including behavioral change, social support, mental health, and preventive health — affects nutrition, physical activity, and stress and CVD risk factor prevalence and control.

Incident cardiovascular disease events and deaths include those from 1) coronary heart disease, 2) stroke, and 3) combined cardiovascular disease, including coronary heart disease, stroke, congestive heart failure, and peripheral arterial disease. These categories are identical to those used by a risk calculator based on the Framingham study (13). Our model modifies the risk calculator somewhat, narrowing its inputs (based on the availability of population survey data) to sex, age, smoking, diabetes, systolic blood pressure, and the ratio of total cholesterol to high density lipoprotein (HDL), but also allowing for "smoking equivalent" risk from secondhand smoke (14) and air pollution (15).

Obesity is not one of the Framingham risk calculator's direct inputs, but the prevalence of obesity is included here as a factor that increases the risks of hypertension, high cholesterol, and diabetes (16). The prevalence of obesity is itself influenced in our model by the quality of nutrition and physical activity. Also, the literature points clearly to adverse direct effects of inadequate physical activity on the onset of hypertension, high cholesterol, and diabetes (17,18). Similarly, poor diet — including insufficient intake of fruits and vegetables and excess intake of salt and saturated and trans fats — has direct effects on the onset of hypertension and high cholesterol (19-22). Chronic stress is another factor that adversely affects blood pressure and cholesterol (23), and it is also known to lead to greater caloric intake (24) and a greater tendency to smoke (25).

How does local context affect these behavioral and environmental drivers of cardiovascular health such as diet, physical activity, smoking, chronic stress, and air pollution, as well as the use of available services for behavioral change, social support, mental health, and preventive health care? We have discussed this question with our team, consulted prior research, and examined local data, including an enhanced version of the Behavioral Risk Factor Surveillance Survey (BRFSS) (26) administered in eastern Travis County. We also compared data from the east and west sides of the county, where possible, with comparable measures for the United States overall. This sort of analysis allowed us to identify suitable metrics for many of the contextual factors in the Figure.

For example, we define chronic stress by reference to two measures: 1) a BRFSS item that asks about getting the social and emotional help one needs and 2) the capacity of local mental health service providers (including psychologists and social workers). On the east side of the county, where fewer social supports and mental health services are available than on the west, a relatively large proportion of people say they do not get the help they need (27% for 2005–2006), whereas on the west side, a much smaller proportion of people say they do not get the help they need (13%). This disparity suggests that the level of chronic stress on the east side could potentially be reduced through expansion of appropriate services and other supports. We do not downplay the importance of poverty, crime, and racial discrimination as sources of chronic stress, but greater access to mental health services and social supports could help mitigate some of the stress experienced in the poorer and more racially mixed area, even while more ambitious efforts are being organized to transform the adverse conditions themselves.

The item furthest upstream in our framework is the local capacity for leadership and organizing, which determines the residents' ability to identify and respond effectively to their own needs through processes such as networking, organizing, fundraising, training, and community action. Local capacity is a well-known concept that is being increasingly better defined and quantified (27-29). As those measures become more widely available, we may use the system dynamics model to analyze whether there are circumstances under which it would be best to invest in building local capacity, even if that means less effort on direct program activities in the short term. A prior exploratory model suggested that this might indeed be the case in areas where local capacity is low and people are challenged by a high and inequitable burden of disease (30).

The concepts depicted in the Figure are currently being brought together in a mathematical model that makes explicit assumptions about the policy options for protecting cardiovascular health. The resulting simulation model will allow planners to address many what-if questions and explore for themselves which strategies hold the greatest potential to reduce the health and economic impacts of cardiovascular risks and events. The model will allow interested stakeholders to see the likely effects of the following hypothetical interventions:

Improving access to, or the effectiveness of, primary care, thereby improving the diagnosis and control of hypertension, high cholesterol, and diabetes.

Improving access to, or the effectiveness of, other types of services (e.g., mental health, smoking cessation, weight loss), thereby reducing the prevalence of stress, smoking, or obesity.

Improving access to affordable and healthy food options and safe spaces for physical activity.

Regulating more strictly smoking in public places and other sources of secondhand smoke and air pollution.

Reducing the sources of chronic stress by addressing issues of poverty, neighborhood safety, and racial discrimination.

Increasing social marketing to encourage healthy behaviors.

Strengthening local public health leadership and organizing capacity for intervention.

## Moving Forward

Our simulation model, like all models, simplifies reality and, therefore, will be incomplete in some ways. Some simplifications will be due to a lack of data, and others will exist for the sake of general applicability. For instance, our current framework does not include some dynamics that are apparent in the real world, such as the social diffusion of behavioral norms, community self-organizing, and market responses to inadequate service levels.

Moreover, even factors included in the model will be subject to some uncertainty about their change over time and the size of their effects. We are attempting to limit these uncertainties through a combination of literature review, data analysis, and expert consensus. Sensitivity tests using the model will then allow us to determine the degree to which remaining uncertainty in our assumptions may affect policy conclusions. This sensitivity testing will identify key areas for further research.

Although the initial phase of theory development has been focused on Austin, we hope that the emerging model will be useful for other settings as well. Toward this end, we have grounded the framework in the broad literature on cardiovascular risk and have attempted to use data that are regularly collected not only in Austin/Travis County but also throughout the nation.

Despite the limitations of simulation models, it is prudent to use them when making policy decisions about inherently complex problems. Intervention trials and observational studies tend to be too narrowly circumscribed to answer broad-reaching policy questions. However, by integrating such evidence along with local experience into a single causal framework and simulation model, conclusions may be reached that are firmer than those based on logic or intuition or simple calculations alone. Despite uncertainties in the data, simulation studies can systematically calculate the net effects of many interrelated factors affecting cardiovascular health and thereby support better policy decisions.
